# A Review of Cardiac Complications in Coronavirus Disease 2019

**DOI:** 10.7759/cureus.8034

**Published:** 2020-05-08

**Authors:** Romil Singh, Rahul Kashyap, Anneka Hutton, Munish Sharma, Salim Surani

**Affiliations:** 1 Internal Medicine, Metropolitan Hospital, Jaipur, IND; 2 Critical Care, Mayo Clinic and Foundation, Rochester, USA; 3 Internal Medicine, Charles E. Schmidt College of Medicine Florida Atlantic University, Boca Raton, USA; 4 Internal Medicine, Corpus Christi Medical Center, Corpus Christi, USA; 5 Internal Medicine, University of North Texas, Dallas, USA

**Keywords:** novel coronavirus, cardiomyopathy, covid 19, heart failure, sars-cov-2

## Abstract

The severe acute respiratory syndrome coronavirus 2 (SARS-CoV-2) infection has shown an association with acute myocardial injury, cardiomyopathy, and myocarditis. Individuals with myocardial involvement in association with the coronavirus disease 2019 (COVID-19) may be at increased risk of developing severe illness. Cardiomyopathies are a heterogeneous group of diseases of the myocardium associated with mechanical and/or electrical dysfunction that usually exhibit inappropriate ventricular hypertrophy or dilation and are due to a variety of causes that frequently are genetic. It has been primarily divided into three subsets: genetic, mixed, and acquired cardiomyopathy. We anticipate that, because of the high inflammatory response, other cardiovascular complications may also occur in COVID-19 patients with severe symptoms. This review explores new information as it pertains to COVID-19 and cardiac complications.

## Introduction and background

The coronavirus disease 2019 (COVID-19) pandemic has affected people worldwide and poses a severe health threat on a global scale. COVID-19 first emerged in Wuhan, Hubei Province, China in December of 2019 with a report of a severe flu-like illness. In January 2020, the causative pathogen was identified as a novel coronavirus, subsequently named SARS-CoV-2 (severe acute respiratory syndrome coronavirus 2). In February 2020, the World Health Organization (WHO) coined the term “COVID-19” in reference to coronavirus disease 2019 [1]. As of April 9th, 2020, over 1,500,000 laboratory-confirmed cases have been reported in 184 countries. Unfortunately, COVID-19 has resulted in over 90,000 deaths [2]. 

Clinical features of COVID-19 typically include fever and respiratory illness of variable severity ranging from cough and dyspnea to respiratory failure and acute respiratory distress syndrome (ARDS). Although much less common, in the existing literature, myocardial injury has been reported anywhere from 19.7% to 29.8% of cases [3]. Furthermore, cases of myocarditis and cardiomyopathy associated with the SARS-CoV-2 infection have emerged in the literature.

Cardiomyopathy is a rare but serious condition that may lead to heart failure. Cardiomyopathy may involve systolic dysfunction, diastolic dysfunction, or both. Systolic dysfunction results in dilated cardiomyopathy (DCM), which has a prevalence of 1:2500 and is the third most common cause of heart failure and the most common cause of heart transplantation, whereas diastolic dysfunction gives rise to restrictive cardiomyopathy, which is associated with infiltrative diseases, storage, or maybe idiopathic [4]. The third phenotypic class is hypertrophic cardiomyopathy (HCM), which carries a prevalence of 1:500 in the absence of aortic valve disease and systemic hypertension [5]. We anticipate that, because of the high inflammatory response, other cardiovascular complications may also occur in COVID-19 patients with severe symptoms. 

## Review

Acute myocardial injury related to COVID-19

SARS-CoV-2 also belongs to the beta-coronavirus subtype similar to coronavirus responsible for severe acute respiratory syndrome (SARS) and the Middle East respiratory syndrome (MERS) [6]. SARS-CoV may have resulted in cardiovascular complications, including acute coronary syndrome and myocardial infarction. SARS-CoV-2 appears to have similar pathophysiology to the coronaviruses responsible for MERS and SARS which also have reports of cardiac involvement [7]. Huang et al. reported that five of 41 patients diagnosed with COVID-19 developed severe myocardial injury manifested as an increase in the high-sensitivity of cardiac troponin I (hs-cTnI) levels (> 28 pg/ml) [8]. 

In a population of 416 patients with COVID-19 infection, Shi et al. reported that 19.7% of patients had a cardiac injury as defined by serum cardiac bio enzymes high-sensitivity troponin I (hs-TnI) above the 99th percentile upper reference limit [3]. Patients with acute myocardial injury tended to be of older age and with an increased number of comorbidities. Based on laboratory abnormalities and radiographic findings, patients with acute myocardial injury tended to have increased severity of illness. Additionally, the degree of respiratory compromise appeared to be more severe. Non-invasive mechanical ventilation was required in 46.3% of patients with acute myocardial injury as compared to 3.9% of those without. Likewise, the need for invasive mechanical ventilation increased in those with cardiac injury compared to those without at 22% and 4.2%, respectively. Importantly, the acute myocardial injury was independently associated with increased mortality of 51.2% compared to 4.5% in those without myocardial injury [3]. 

In their analysis of six studies inclusive of 1,527 patients, Li et al. examined the association with COVID-19 and cardiovascular disease (CVD) [9]. Among hospitalized patients with confirmed SARS-CoV-2 infection, the prevalence of cardiac and cerebrovascular disease, hypertension, and diabetes was reported to be 16.4%, 17.1%, and 9.7%, respectively. Li et al. additionally reported that 8% of COVID-19 patients suffered from an acute cardiac injury. Furthermore, the incidence of acute cardiac injury was approximately 13-fold higher in patients who were critically ill and required intensive care unit admission (ICU) compared with those who were not critically ill [9]. Similarly, Zhou et al. described the acute myocardial injury as the most common cardiovascular complication with an incidence of 8% - 12% on average which either resulted from direct myocardial injury, systemic inflammation, myocardial oxygen demand-supply mismatch, acute coronary event, or iatrogenic injury [10]. Table 1 summarizes the current literature for acute myocardial injury in COVID 19.

**Table 1:** Summary of Literature for Acute Myocardial Injury in COVID-19 CK-MB: creatine kinase myocardial band; COVID-19: coronavirus disease 19; NT-proBNP: N-terminal pro-brain natriuretic peptide

Author	Study Design	Sample Size	Findings
Guo et al. [11]	Case Series	187 COVID-19 patients with a mean age of 58.5 years	52 patients suffered an acute myocardial injury with a mean CK-MB fraction of 3.34 ng/ml; mean myoglobin of 128.7 μg/ml
Li et al. [9]	Meta-analysis	A total of six studies with 1,527 COVID-19-positive patients were included in this analysis	122 patients (8%) showed signs of acute cardiac injury
Zhou et al. [10]	Retrospective Cohort Study	191 COVID-19-positive patients with a mean age of 56 years (range: 46–67) were included.	33 patients (17%) suffered from acute cardiac injury out of which 24 patients had a high sensitivity cardiac troponin I level of > 28 pg/ml.
Huang et al. [8]	Case Series	41 COVID-19-positive patients with a mean age of 49 years (range: 41-58) were included	5 patients (12%) were diagnosed with acute myocardial injury with an increase in troponin I level of >28 pg/ml.
Inciardi et al. [12]	Case Report	A 53-year-old woman who tested positive for COVID-19 was admitted to the cardiac care unit in March 2020 for acute myopericarditis with systolic dysfunction confirmed on cardiac magnetic resonance imaging, the week after onset of fever and dry cough due to COVID-19	Findings included high-sensitivity troponin T level of 240 pg/ml and CK-MB level of 20.3 ng/mL with elevated NT-proBNP levels (5,647 pg/mL)

Prevalence of cardiomyopathy and subsequent heart failure in COVID-19 patients

In a case series involving 187 SARS-CoV-2-positive patients, 27.8% had a myocardial injury resulting in cardiac dysfunction and arrhythmia [11]. The development of acute myocardial injury has been associated with an increased risk of developing cardiogenic shock and malignant arrhythmias. Furthermore, 4.3% (8/187) had cardiomyopathy [11]. In their study of 21 critically ill patients with reverse transcription-polymerase chain reaction (RT-PCR)-confirmed COVID-19, Arentz et al. reported the development of cardiomyopathy in 33% (7/21) of patients [13]. 

Inciardi et al. reported the case of a patient with COVID-19 who developed myocarditis [12]. Troponins were found to be elevated and echocardiography revealed diffuse hypokinesis and a left ventricular ejection fraction of 40%. Cardiac magnetic resonance imaging (MRI) showed diffuse biventricular myocardial interstitial edema and late gadolinium enhancement was in line with acute myocarditis.

The underlying pathogenesis of COVID-19 myocarditis is currently unclear; theories include inflammation caused by an exaggerated immune response or damage due to direct viral injury. To the best of our knowledge, the viral genome has not yet been isolated from cardiac tissue. This may suggest a direct viral infection of the myocardium. In patients infected with SARS-CoV, the viral genome was detected in their cardiac tissue in 35% of cases. As SARS-CoV and SARS-CoV-2 share a very homologous genome, direct viral invasion of the myocardium is likely possible [14]. Furthermore, SARS-CoV-2 infection occurs through receptor-mediated endocytosis triggered by binding of the viral spike protein to the angiotensin-converting enzyme 2 (ACE-2) receptor. Rich expression of ACE-2 within lung alveolar cells is thought to be the mechanism by which the respiratory tract serves as the main entry for viral infection. The ACE-2 receptor is also highly expressed in cardiac tissue and likewise may serve as the vehicle for viral invasion into the myocardium [9, 15].

Similarities also exist between the pathogenicity of MERS-CoV and SARS-CoV-2. A prior case study revealed an association of MERS-CoV and the development of myocarditis and heart failure [7]. The estimated prevalence of COVID-19 associated cardiomyopathy varies widely. Precise estimates of prevalence are limited by the unavailability of widespread testing, variations in national surveillance, and lack of standardized data collection.

Cardiomyopathy may lead to the development of heart failure syndrome. Zhou and his team reported that 23.0% of patients with COVID-19 presentations are characterized by heart failure [10]. Interestingly, the development of heart failure syndrome was more commonly observed than acute kidney injury. It is unclear whether heart failure in COVID-19 occurs due to the development of new cardiomyopathy or exacerbation of preexisting left ventricular dysfunction [8]. Table 2 summarizes the findings of the current literature regarding cardiomyopathy in COVID-19.

**Table 2:** Summary of Literature for Cardiomyopathy and Heart Failure in COVID-19 COVID-19: coronavirus disease 2019; NT-pro-BNP: N-terminal pro-brain natriuretic peptide

Author	Study design	Sample size	Findings
Arentz et al. [13]	Case Series	21 critically ill patients with COVID-19 were included with a mean age of 70 years (range: 43-92 years)	7 patients (33%) developed cardiomyopathy
Guo et al. [11]	Case Series	187 COVID-19 patients with a mean age of 58.50 years.	8 patients (4.3%) developed cardiomyopathy with a mean elevated troponin T level of 15.4 pg/ml; mean NT-proBNP was 817.4 pg/ml
Zhou et al. [10]	Retrospective Cohort Study	191 COVID-19-positive patients with a mean age of 56 years (46–67) were included.	44 patients (23%) developed heart failure.

Other cardiac manifestations of COVID-19 

**Figure 1:**
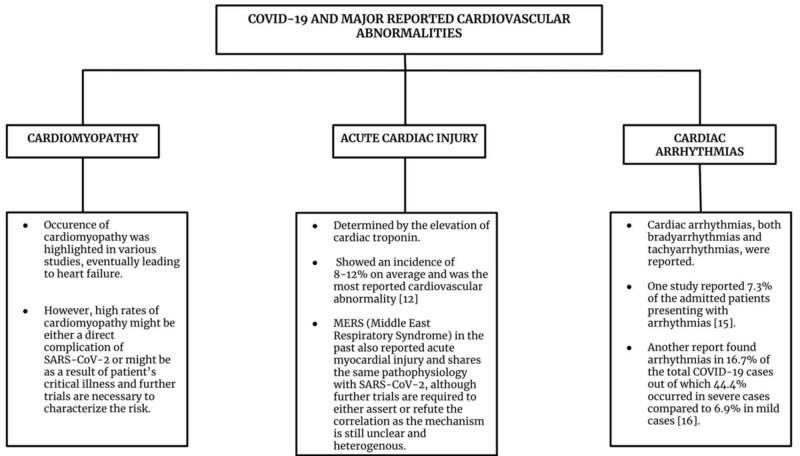
Summary of COVID-19 and major cardiovascular abnormalities COVID-19: coronavirus disease 19; SARS-CoV-2: severe acute respiratory syndrome coronavirus 2

Aside from cardiomyopathy and acute myocardial injury, further cardiovascular involvement in COVID-19 includes cardiac arrhythmia. Liu et al. reported that 10 out of 137 patients admitted for COVID19 disease, cardiac arrhythmia was part of the presenting symptomatology [15]. Cardiac arrhythmia was also noted in 34 out of 138 patients and was more common in ICU patients compared to non-ICU patients (44.4% vs. 6.9%) [16]. The high prevalence of arrhythmia was suggested to be attributable to hypoxia, metabolic disarray, or neurohormonal stress in the setting of viral infection [17]. Figure 1 summarizes the major cardiovascular findings in COVID-19. The mortality, once set in, is very high in cardiac patients with COVID-19. The preliminary experience is for early intubation in patients who have cardiac involvement and COVID-19 going into respiratory distress to avoid acute hemodynamic respiratory compromise. The authors will await recommendations based on emerging data.

## Conclusions

SARS-CoV-2 is an emerging infection of global importance. Much remains to be understood about the disease and the complications caused by this virus, such as myocardial injury. Myocardial injury is associated with inflammation, impairment of cardiac function, and malignant ventricular tachyarrhythmias. When COVID-19 is complicated by acute myocardial injury, a significant increase in mortality occurs. Furthermore, this novel disease may be complicated by myocarditis and heart failure. We urge physician awareness of the elevated morbidity and mortality of myocardial involvement in COVID-19. In the coming months, efforts towards the identification of effective therapies will be crucial in improving outcomes in the critically ill patient.
